# Di-n-Butyl Phthalate and Its Monoester Metabolite Impairs Steroid Hormone Biosynthesis in Human Cells: Mechanistic In Vitro Studies

**DOI:** 10.3390/cells11193029

**Published:** 2022-09-27

**Authors:** Liselott Källsten, Paula Pierozan, Jonathan W. Martin, Oskar Karlsson

**Affiliations:** Science for Life Laboratory, Department of Environmental Science, Stockholm University, 114 18 Stockholm, Sweden

**Keywords:** anti-androgenic, cortisol, endocrine disruptor, EDC, oxidative stress, ROS, steroidogenesis, testosterone

## Abstract

The widespread environmental contaminant di-n-butyl phthalate (DBP) has been linked with reduced testosterone levels and adverse reproductive health outcomes in men. However, the underlying mechanisms of these anti-androgenic effects and the potential effects on other classes of steroid hormones remain to be elucidated. Here, we conducted mechanistic studies in human adrenocortical H295R cells exposed to 1–500 µM of DBP or its metabolite, mono-n-butyl phthalate (MBP), for 48 h. Quantification of steroid hormones in the cell medium by liquid chromatography-mass spectrometry revealed that both phthalates significantly decreased testosterone, androstenedione, corticosterone, and progesterone levels, in particular after dibutyryl-cyclic-AMP stimulation of steroidogenesis. Western blot analysis of key steroidogenic proteins showed that DBP induced a dose-dependent decrease of CYP11A1 and HSD3β2 levels, while MBP only significantly decreased CYP17A1 levels, indicating that the compounds affect early steps of the steroidogenesis differently. Both DBP and MBP exposure also lead to a dose-related decrease in HSD17β3, the enzyme which catalyzes the final step in the testosterone biosynthesis pathway, although these effects were not statistically significant. Interestingly, DBP increased the cortisol concentration, which may be due to the non-significant CYP11B1 increase in DBP-exposed cells. In contrast, MBP decreased cortisol concentration. Moreover, the analysis of superoxide generation and quantification of the protein oxidation marker nitrotyrosine demonstrated that DBP induced oxidative stress in H295R cells while MBP reduced protein nitrotyrosine levels. These findings confirm the anti-androgenic effects of DBP and MBP and reveal several differences in their toxicological mechanisms, with possible implications for future research on phthalate toxicity.

## 1. Introduction

Phthalates are synthetic chemicals found in numerous commercial products and materials, mainly as plasticizers [1]. Phthalates can also be used as additives in nail polish, perfumes, drug coatings, and even skin creams [1,2]. Because of this ubiquitous use, phthalates are easily emitted or leached from products and are widespread in the environment [3,4]. Di-n-butyl phthalate (DBP) is one of the most commonly used phthalates, and it is frequently detected in food [1] and human samples [5]. In addition, DBP is among the phthalates with the most evident anti-androgenic effects, both from epidemiological [5] and in vivo studies [6,7].

While previous studies investigate the mechanism behind these anti-androgenic effects in vivo in mouse models [8] and in vitro in rat and murine Leydig cells [9,10,11], there is currently a lack of mechanistic studies using human model systems. Furthermore, in humans and animals, ingested DBP is metabolized by esterases in the intestine to mono-n-butyl phthalate (MBP) [12,13], which is also considered to be biologically active [9,14]. However, most in vitro mechanistic studies use either DBP or MBP. To our knowledge, only a few published studies compare their mechanism of action, and none of them are in human cells [9,10].

**Figure 1:**
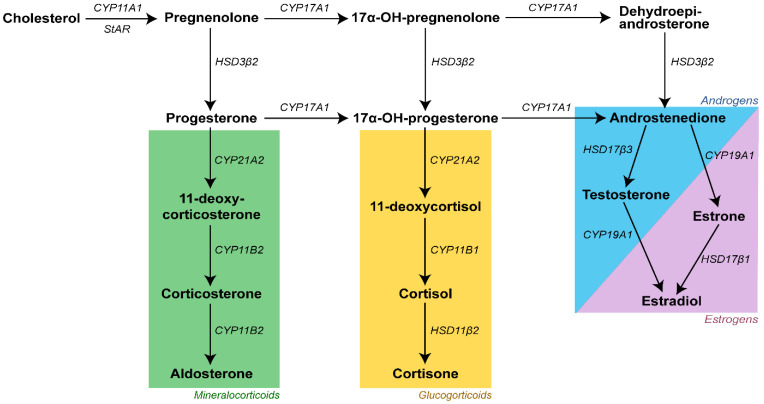
Overview of the steroid hormone synthesis in H295R cells. Hormones are written in bold, and the enzymes catalyzing the different steps are written in italics. Adapted from Jeanneret et al., 2016 [21].

The H295R cells are a suitable model system to study the effects of environmental contaminants on steroidogenesis, including underlying mechanisms. These cells originate from the parent NCI-H295 cell line, which was established from adrenocortical carcinoma cells [15]. H295R cells have all of the adrenocortical steroidogenic enzyme systems intact, which gives them a unique ability to produce all steroid hormones normally produced by the different zones of the adrenal gland, including androgens and estrogens (Figure 1) [15,16,17]. This cell line is also one of the few established models to study steroidogenic disruption in human cells. The other cell line options are all granulosa cells and therefore not as suitable to study anti-androgenic effects [18]. Two previous studies have been published with H295R cells exposed to DBP or MBP. One study demonstrated that both DBP and MBP could induce decreased testosterone production in this cell line [19]. The other study focused on the effects on cortisol secretion after phthalate exposure and observed no changes after 48 h exposure to 30 µM DBP [20]. However, neither of these studies provides any mechanistic data.

The aim of the current study was to examine and compare how DBP and its main metabolite, MBP, affect the steroid hormone biosynthesis, including key steroidogenic enzymes, and certain parameters of oxidative stress, using the human cell line H295R as an in vitro model. This study also examined if stimulating the steroidogenesis with dbcAMP affects how the cells respond to phthalate exposure.

## 2. Methods and Materials

### 2.1. Chemicals

DBP (CAS No 84-74-2, purity > 99%), MBP (CAS No 131-70-4, purity > 99%), analytical grade standards of testosterone, androstenedione, corticosterone, cortisol and progesterone, isotope-labeled testosterone-d3, corticosterone-d4 and progesterone-^13^C5, SIGMAFAST™ protease inhibitor tablets, 3-(4,5-Dimethylthiazol-2-yl)-2,5-Diphenyltetrazolium Bromide (MTT), dimethyl sulfoxide (DMSO), and dbcAMP were all purchased from Sigma Aldrich (St. Louis, MO, USA). Dulbecco’s modified Eagle medium/HamF12 (DMEM/F12), FluoroBrite™ DMEM, Dulbecco’s Phosphate Buffered Saline (DPBS), and trypsin-EDTA were purchased from Gibco Life Technologies Europe (Bleiswijk, the Netherlands). Insulin-Transferrin-Selenium (ITS+) premix and Nu-serum were purchased from Corning Discovery Labware, Inc. (Bedford, MA, USA). Superblock™ blocking buffer was purchased from Thermo Scientific (Rockford, IL, USA). Tween 20, NaCl and Tris-HCl, LC-MS grade water and methanol were purchased from Fisher (Longborough, UK). Ammonium fluoride was purchased from Honeywell Research Chemicals (Morris Plains, NJ, USA). Triton X-100 was purchased from VWR international (Radnor, PA, USA). No-Stain™ Protein Labeling Reagent (Cat.no. A44717), dihydroethidium (DHE), cumene hydroperoxide, and Hoechst 33342 were purchased from Invitrogen, Life Technologies Corp. (Carlsbad, CA, USA). TBS was purchased from Medicago AB (Uppsala, Sweden). Sodium deoxycholate was purchased from Alfa Aesar ThermoFisher GmbH (Kandel, Germany). Dry milk was purchased from Cell signaling Technology (Danvers, MA, USA). The chemiluminescence ECL kit was purchased from Bio-Rad (Hercules, CA, USA).

### 2.2. Cell Culture

H295R cells were obtained from the American Type Culture Collection (ATCC, Manassas, VA, USA). The cells were cultured and maintained according to the OECD guideline 456. In brief, the cells were maintained as a monolayer in 75 cm^2^ tissue culture flasks with 20 mL culture medium. The culture medium consisted of DMEM-F12 medium supplemented with 2.5% Nu-serum and 1% ITS+ premix. All incubations were done at 37 °C with 5% CO_2_.

### 2.3. Steroidogenesis Assay and Viability Test

Cells were seeded in 96-well plates at a density of 50,000 cells/well in 200 µL culture medium. After 24 h incubation, the medium was replaced with new medium containing DBP or MBP added at concentrations of 0 (0.1% DMSO, solvent control), 1, 10, 100, or 500 µM. Forskolin (1 µM) and prochloraz (0.3 µM) were added as the positive and negative control, respectively, for the steroidogenesis assay. For each replicate, one plate was prepared with a standard culture medium and one plate with the addition of 0.1 mM dbcAMP to the medium to stimulate steroidogenesis. After 48 h exposure to the test compounds, the medium was collected and stored at −80 °C until analysis. The removed medium was then replaced with 100 µL/well of MTT (0.5 mg/mL) dissolved in DMEM-F12. After 45 min incubation at 37 °C, the MTT solution was removed, and the formazan generated was solubilized by adding 50 µL DMSO to each well and leaving the plate on a plate shaker at 500 rpm for 10 min. The absorbance of the dissolved formazan was measured at 570 nM using a SpectraMax i3 microplate reader (Molecular Devices, San Jose, CA, USA).

### 2.4. Quantification of Steroid Hormones

The collected culture medium was thawed, diluted 1:2 with methanol and left on a plate shaker at 500 rpm for 20 min to precipitate proteins. The plate was then centrifuged at 2500× *g* for 15 min. The supernatant was filtered with a 96-well filter plate (0.2 µM, PTFE filter; Agilent, Santa Clara, CA, USA) by centrifugation at 3000× *g* for 10 min. The filtrate was then analyzed by liquid chromatography-mass spectrometry (LC-MS). Progesterone, corticosterone, androstenedione, and testosterone were quantified as previously described [8], with the only modification that cortisol was added to the method. In brief, chromatographic separation was achieved using an Ultimate 3000 ultra-high pressure LC system (Thermo Scientific, Sunnyvale, CA, USA) with an Acquity UPLC BEH C18 analytical column (2.1 × 50 mm, 1.7 µm particles) and VanGuard Acquity UPLC BEH C18 pre-column (2.1 × 5 mm, 1.7 µm particles; Waters, Milford, MA, USA). The mobile phases were water with 1 mM ammonium fluoride, and methanol. Mass spectral data were acquired with a Q-Exactive HF-X Orbitrap (Thermo Fisher Scientific, Bremen, Germany) high-resolution mass spectrometer (HRMS), employing electrospray ionization in positive mode. Data were acquired in full scan mode (MS1) in parallel with a targeted PRM (MS2) employed for the analytes progesterone, corticosterone, cortisol, androstenedione, and testosterone, and the internal standards testosterone-d3, corticosterone-d4, and progesterone-^13^C5.

### 2.5. Levels of Steroidogenic Enzymes

The levels of nine key steroidogenic proteins were measured by western blot. Cells were cultured in 6-well plates at 500,000 cells/well. After attaching to the plate for 24 h, the cells were exposed to 10 and 100 µM DBP, 100 and 500 µM MBP, or solvent control (0.1% DMSO) dissolved in a culture medium with 0.1 mM dbcAMP. After 48 h exposure, the cells were washed with DPBS and trypsinized for 10 min. The detached cells were then collected in tubes and lysed with RIPA buffer (150 mM NaCl, 0.1% Triton X-100, 0.5% sodium deoxycholate, 0.1% SDS, 50 mM Tris-HCl, 1% protease inhibitors pH 8). Protein concentration in the lysate was determined by the Lowry assay [22], and 20 µg of protein per sample was separated by SDS-PAGE on a 4–20% gel before being transferred to nitrocellulose membranes (Bio-Rad, Hercules, CA, USA). Total protein content in each lane was measured by staining with a No-Stain™ Protein Labeling Reagent kit according to the manufacturer’s protocol. The membranes were then blocked for 1 h in TBS with 5% dry milk and incubated overnight with primary antibodies (Table 1), diluted in Superblock™ solution. After the incubation, the membranes were washed twice with TBS-Tween (TTBS) and twice with TBS. Secondary antibody (peroxidase-conjugated goat anti-rabbit or goat anti-mouse (Cat. no. 5196-2504 and 0300-0108P respectively, Bio-Rad, Hercules, CA, USA)) diluted 1:5000 in Superblock™ was then added, and the blots were incubated for 1 h, followed by washes as described above. The blots were developed with an ECL-kit using a charge-coupled device (CCD) imager (iBright 1500 Imaging System; ThermoFisher, Rockford, IL, USA), and the optical density for the blots and total protein staining were measured with ImageJ ver 1.53r (NIH, Bethesda, MD, USA). The results were normalized against the total protein content for each sample.

### 2.6. Analysis of Oxidative Stress

Cells were seeded in black 96-well plates with a transparent bottom at a density of 25,000 cells/well, either in a normal culture medium or in a medium with 0.1 mM dbcAMP added, and cultured for 48 h. The cells were then exposed for 2 h to 1, 10, 100 or 500 µM MBP; 1, 10 or 100 µM DBP, or 0.1% DMSO (solvent control). Since reactive oxygen species (ROS) such as superoxide (˙O_2_^−^) are highly reactive and have a short half-life, a shorter exposure time was selected based on pilot studies. Positive control was 1 h exposure to 25 µM cumene hydroperoxide. At the end of the exposure, the medium was changed to the staining solution, which consisted of 15 µM of the fluorogenic probe DHE that reacts strongly with ˙O_2_^−^ and to a lesser extent with other ROS and NO, and 5 µg/ mL Hoechst 33342 dissolved in FluoroBrite DMEM, as normal DMEM-F12 has high autofluorescence that can interfere with the analysis. The cells were stained for 40 min, washed twice with DPBS, and then left in FluoroBrite medium for imaging. Images were acquired at 10× magnification from four sites per well using an ImageXpress Micro^®^ Confocal High-Content Analysis System (Molecular Devices, San Jose, CA, USA). ImageJ ver 1.53r (NIH, Bethesda, MD, USA) was used to count the number of DHE and Hoechst stained nuclei. Results are reported as the number of DHE-stained cells divided by the number of Hoechst-stained cells.

### 2.7. Statistics

Results are presented as mean ± standard deviation (SD) for each experiment. Differences compared to the control were analyzed by one-way analysis of variance (ANOVA) followed by Dunnet’s multiple comparison test for western blot results and by a one-sample *t*-test against a hypothetical mean of 100 for the other experiments, where the average was first calculated for each replicate and then combined. All statistical tests were done using Prism 9 (GraphPad Software, San Diego, CA, USA).

## 3. Results

### 3.1. Cell Viability

**Figure 2:**
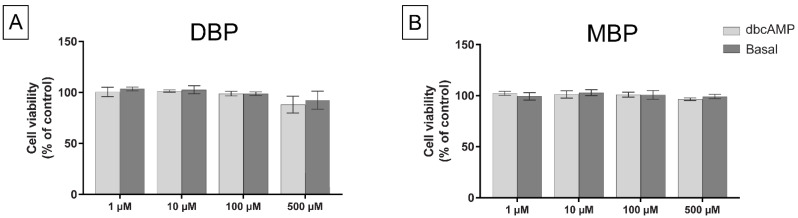
The MTT assay was used to measure cell viability in H295R cells after 48 h exposure to either solvent control (0.1% DMSO), DBP (**A**) or MBP (**B**), with or without stimulation of the steroidogenesis by 0.1 mM dbcAMP. Values represent mean ± S.D. calculated as % of control for three replicates, where the average of six wells/treatment was calculated for each replicate (One sample *t*-test).

The viability of the cells after 48 h phthalate exposure was determined by the MTT assay. No significant cell death was observed for any of the DBP (Figure 2A) or MBP (Figure 2B) exposures. However, a non-significant decrease (88% of control, *p* = 0.06) was observed at 500 µM DBP with 0.1 mM dbcAMP added to the cell medium, indicating that this concentration might have some cytotoxic effects. The same concentration without stimulation had less effect (92% of control, *p* = 0.14).

### 3.2. Steroid Hormone Levels

**Figure 3:**
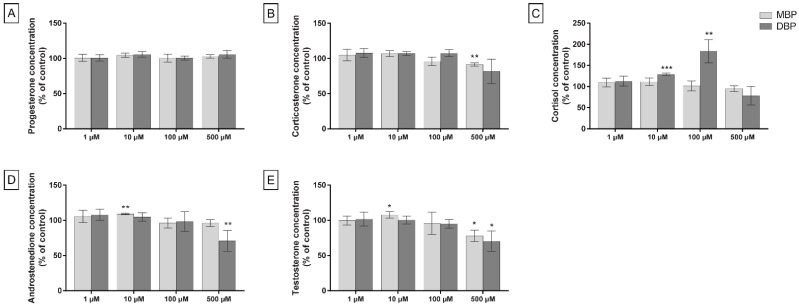
H295R cells exposed to DBP, MBP, or solvent control (0.1% DMSO) for 48 h show altered levels of steroid hormones. Levels of progesterone (**A**), corticosterone (**B**), cortisol (**C**), androstenedione (**D**) and testosterone (**E**) were measured in the cell medium by LC-MS. Values represent mean ± S.D. calculated as % of control for three replicates, where the average of six wells/treatment was calculated for each replicate. Statistically significant differences from a theoretical mean of 100 are indicated as follows: * *p* < 0.05, ** *p* < 0.01, *** *p* < 0.001 (One Sample *t*-test).

The levels of five steroid hormones were measured by LC-MS in the cell medium after 48 h of phthalate exposure. When the cells were cultured in standard cell culture medium, no changes were observed in progesterone concentration after DBP or MBP exposure (Figure 3A). Both compounds decreased the levels of corticosterone at the highest concentration (DBP: 18% decrease, *p* = 0.12; MBP: 8.4% decrease, *p* = 0.005), but it was significant only for MBP (Figure 3B). The levels of cortisol demonstrated the largest difference in effects between DBP and MBP (Figure 3C). DBP induced a significant increase of cortisol at 10 and 100 µM (29% (*p* = 0.0003) and 84% (*p* = 0.008) increase, respectively), while MBP induced a slight and non-significant decreasing trend (*p* = 0.25 at 500 µM, Figure 3C). DBP exposure induced a significant decrease of androstenedione (29% decrease, *p* = 0.009) and testosterone (30% decrease, *p* = 0.03) at 500 µM. While an overall decreasing trend could also be observed after MBP exposure, with a significant effect for testosterone (22% decrease, *p* = 0.01) at 500 µM, a small but significant increase in androstenedione and testosterone levels (9% (*p* = 0.006) and 8% (*p* = 0.04) increase, respectively) were observed at 10 µM MBP (Figure 3D–E).

**Figure 4:**
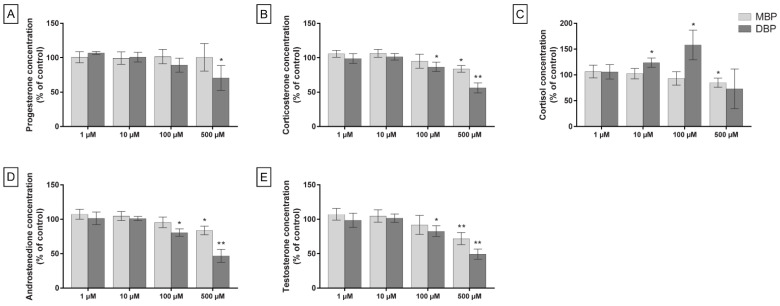
H295R cells exposed to DBP, MBP, or solvent control (0.1% DMSO) for 48 h, with stimulation of the steroidogenesis by 0.1 mM dbcAMP, show altered levels of steroid hormones. Levels of progesterone (**A**), corticosterone (**B**), cortisol (**C**), androstenedione (**D**), and testosterone (**E**) were measured in the cell medium by LC-MS. Values represent mean ± S.D. calculated as % of control for three replicates, where the average of six wells/treatment was calculated for each replicate. Statistically significant differences from a theoretical mean of 100 are indicated as follows: * *p* < 0.05, ** *p* < 0.01 (One Sample *t*-test).

When the cells were exposed to the phthalates under stimulated conditions (0.1 mM dbcAMP), the trends were similar, but with overall larger effects for all the quantified hormones, except progesterone, where MBP still had no effect, but a significant decrease in hormone levels could now be observed at 500 µM DBP (29% decrease, *p* = 0.048; Figure 4A). MBP exposure at 500 µM significantly decreased the corticosterone (22%, *p* = 0.03), androstenedione (22%, *p* = 0.03), and testosterone (30%, *p* = 0.005) levels, while DBP exposure induced a significant decrease already at 100 µM for these hormones (17% (*p* = 0.04), 25% (*p* = 0.02) and 20% (*p* = 0.02) decrease, respectively; Figure 4B,D,E). DBP still induced a significant increase in cortisol concentration at 10 and 100 µM (19% (*p* = 0.04) and 56% (*p* = 0.02), respectively; Figure 4C). However, this effect was less prominent compared to the effect after exposure without stimulation. On the other hand, MBP significantly decreased the levels of cortisol at 500 µM (19% decrease, *p* = 0.04; Figure 4C).

Both at basal conditions and when the steroidogenesis in the cells was stimulated with dbcAMP, DBP induced larger effects than MBP on all quantified hormones (Figure 3 and Figure 4). After DBP exposure, the largest effects were the increase in cortisol at 100 µM DBP in a standard medium and the decrease of androstenedione and testosterone at 500 µM DBP under stimulated conditions (49% (*p* = 0.003) and 46% (*p* = 0.004) decrease, respectively). The largest effect of MBP exposure was, in comparison, a less prominent decrease in testosterone at 500 µM MBP and 0.1 mM dbcAMP co-treatment (30% decrease, *p* = 0.005).

### 3.3. Levels of Steroidogenic Proteins

To examine the mechanisms behind the demonstrated changes in steroid hormone concentrations, the levels of nine key steroidogenic proteins were measured by western blot: steroidogenic acute regulator (StAR), cytochrome P450 (CYP) 11A1, hydroxysteroid dehydrogenase (HSD) 3β2, CYP17A1, CYP21A2, CYP11B2, CYP11B1, HSD11β2, and HSD17β3. Since the changes in hormone levels were mostly larger under stimulated conditions, these experiments were all conducted with 0.1 mM dbcAMP added to the medium. The highest DBP concentration included for this analysis was 100 µM, which was the lowest concentration that induced significantly altered steroid levels. Since MBP showed less potent effects on steroid levels, the 500 µM concentration was kept as the highest concentration for the western blot analysis of steroidogenic proteins.

**Figure 5:**
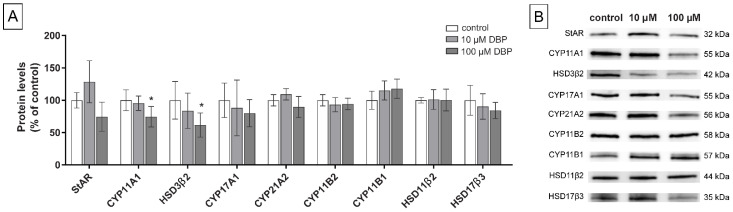
Levels of nine different steroidogenic proteins were measured by western blot (**A**) in H295R cells exposed to 0 (0.1% DMSO, solvent control), 10, or 100 µM DBP for 48 h, with stimulation of the steroidogenesis by 0.1 mM dbcAMP. Values represent mean ± S.D. from six replicates per treatment. Representative blots for each protein are shown (**B**). Total protein stain was used as a loading control. The statistically significant differences from the control are indicated as follows: * *p* < 0.05 (One-way ANOVA followed by Dunnet’s test).

**Figure 6:**
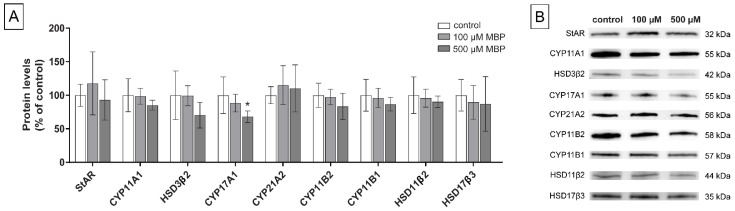
Levels of nine different steroidogenic proteins were measured by western blot (**A**) in H295R cells exposed to 0 (0.1% DMSO, solvent control), 100, or 500 µM MBP for 48 h, with stimulation of the steroidogenesis by 0.1 mM dbcAMP. Values represent mean ± S.D. from six replicates per treatment. Representative blots for each protein are shown (**B**). Total protein stain was used as a loading control. The statistically significant differences from control are indicated as follows: * *p* < 0.05 (One-way ANOVA followed by Dunnet’s test).

Cells exposed to DBP for 48 h showed decreased levels in a dose-response manner with significant effects at 100 µM DBP for two enzymes: CYP11A1 (*p* = 0.02), and HSD3β2 (*p* = 0.04). A similar, but not statistically significant, decreasing trend could be observed for the levels of CYP17A1 (*p* = 0.45) and HSD17β3 (*p* = 0.28). DBP also induced a slight, but not significant, increase in CYP11B1 levels (*p* = 0.09). No changes or trends were observed for the remaining measured proteins (Figure 5A). In comparison, although tested at higher concentrations, MBP only induced significantly decreased levels of one enzyme: CYP17A1 (*p* = 0.02). A non-significant dose-related decreasing trend was observed for CYP11A1 (*p* = 0.23), HSD3β2 (*p* = 0.10), CYP11B2 (*p* = 0.20), HSD11β2 (*p* = 0.56) and HSD17β3 (*p* = 0.69). No trend was observed for StAR or CYP21A2 (Figure 6A).

### 3.4. Oxidative Stress

**Figure 7:**
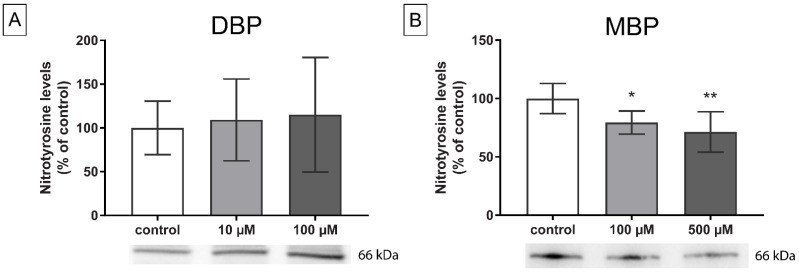
Changes in nitrotyrosine levels in H295R cells after 48 h exposure to DBP (**A**) or MBP (**B**), with stimulation of the steroidogenesis by 0.1 mM dbcAMP, were measured by western blot. Representative blots are shown. Total protein stain was used as a loading control. Values represent mean ± S.D. from six wells per concentration. Statistically significant differences from control are indicated as follows: * *p* < 0.05, ** *p* < 0.01 (One-way ANOVA followed by Dunnet’s test).

**Figure 8:**
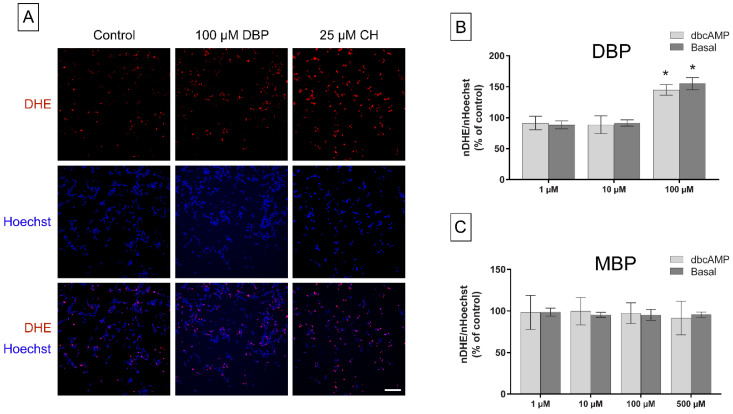
Superoxide levels were measured in H295R cells after 2 h exposure to DBP or MBP. Representative images of cells stained with Hoechst (blue) or DHE (red) and composite images are shown of cells exposed to solvent control (0.1% DMSO), 100 µM DBP, or 25 µM cumene hydroperoxide (CH, positive control; (**A**). Changes in superoxide levels after 2 h exposure to DBP (**B**) or MBP (**C**), with or without stimulation of the steroidogenesis by 0.1 mM dbcAMP, were calculated as the number of DHE-stained objects per image divided by the number of Hoechst-stained objects. Values represent mean ± S.D. calculated as % of control for three replicates, where the average of six wells/treatment was calculated for each replicate. Statistically significant differences from a theoretical mean of 100 are indicated as follows: * *p* < 0.05 (One Sample *t*-test). Scale bar = 200 µM.

Previous studies show that both DBP and MBP can induce oxidative stress in steroidogenically active tissues in vivo [8,23,24]. Two different experiments were conducted to measure the effect of DBP and MBP exposure on oxidative stress in H295R cells: western blot analysis of protein nitrotyrosine levels, and staining with the fluorogenic probe DHE, a marker for superoxide production. DBP induced no significant changes in the protein nitrotyrosine levels, a marker for protein oxidation, after 48 h exposure under dbcAMP stimulated conditions, although a slightly increasing trend was observed (*p* = 0.81; Figure 7A). In contrast, 48 h exposure to both 100 and 500 µM MBP induced a significant decrease in nitrotyrosine levels (*p* = 0.04 and *p* = 0.004 respectively; Figure 7B). Cells exposed to 100 µM DBP for 2 h had a significant increase in the number of nuclei with a positive signal for DHE, with similar effects observed regardless of whether steroidogenesis was stimulated or not (*p* = 0.01 for both conditions; Figure 8A,B). No changes compared to the control were observed after MBP exposure (Figure 8C).

## 4. Discussion

Man-made endocrine-disrupting chemicals in our environment are an increasing problem in modern society. Several epidemiological studies and data from in vivo and in vitro rodent models link exposure to the environmental contaminant DBP with decreased testosterone levels [5,6]. In this study, we examined the mechanisms behind the reduced testosterone production in a human cell model. We show that both DBP (100–500 µM) and MBP (500 µM) can reduce the levels of corticosterone and androstenedione, in addition to testosterone, in H295R cells, especially when steroidogenesis is stimulated with dbcAMP, and that MBP is the less potent compound. Only DBP (500 µM) induced a decrease in progesterone levels, and only under stimulated conditions. Interestingly, DBP induced an increase in cortisol production and superoxide levels, while MBP decreased the cortisol concentration and reduced the levels of the protein oxidation marker nitrotyrosine. Differences could also be observed in how DBP and MBP affected the levels of key steroidogenic proteins.

This study confirms the results from a study by Duan and colleagues, where DBP reduced testosterone levels in H295R cells at lower concentrations compared to MBP. Notably, they observed significantly reduced concentrations already at 0.25 µM DBP and 25 µM MBP [19]. However, as they found significantly reduced cell viability at the lowest concentration included in their viability assay (100 µM), it cannot be excluded that the viability was also decreased at the exposure levels used for the steroidogenesis assay. These discrepancies in potency could be due to the fact that the steroid synthesis in their cells was continuously stimulated by the addition of 0.1 mM dbcAMP, whereas the cells in our study were only stimulated during the time of phthalate exposure [19]. Another study that compared the effect of DBP and MBP in vitro, although in murine Leydig cells, also observed that DBP reduced testosterone levels more potently and induced more significant decreases in the activity of several steroidogenic enzymes. However, MBP was more potent at reducing the levels of the testosterone metabolite 5α-androstanediol [10]. The small but significant increase in testosterone and androstenedione levels observed in our study after exposure to 10 µM MBP could indicate a non-monotonic dose response. However, the effect is minor and might not be biologically relevant, especially considering that the same trend is not observed for the other hormones.

The production of steroid hormones in the adrenal gland is stimulated by the adrenocorticotropic hormone (ACTH), which is produced by the pituitary. Previous studies have demonstrated that the H295R cells have a low response to ACTH and that forskolin or dbcAMP can be used instead to mimic this stimulation [17,25]. The fact that the phthalate-induced decrease of testosterone, corticosterone, and androstenedione was more substantial, and that progesterone was exclusively affected, under stimulated conditions could indicate that adrenal cells are more sensitive to DBP and MBP toxicity under conditions of stress and certain disease conditions with overactive steroid synthesis. This is important as humans are continuously exposed to many environmental contaminants and other stressors that can induce adverse effects, independently or when interacting [26].

Epidemiological data has, in addition to the anti-androgenic effects, linked increased levels of phthalates in urine with increased concentrations of urinary cortisol levels [27]. Interestingly, our results revealed that exposure of H295R cells to 10 and 100 µM DBP induced a significant increase in the cortisol concentration, which may be due to the increased CYP11B1 levels, although this effect was not statistically significant. On the other hand, exposure to 500 µM MBP decreased the cortisol levels in the cells. Only a few previous studies have examined the potential effects of DBP and its metabolite on corticosterone or cortisol biosynthesis. A recent study in 6–8 weeks-old rats observed decreased corticosterone levels in serum after two weeks of exposure to DBP at doses of 500 mg/kg/day or higher [28]. In contrast, the in vivo data from our recent study where adult male mice were exposed to 10 or 100 mg/kg/day DBP for five weeks revealed no significant changes in corticosterone concentrations when measured one week after the last oral administration [8]. These discrepancies could suggest that the effects on the corticosteroid biosynthesis can be reversible, or could be due to differences between species, in the dose and duration of the exposure, where the mechanism of action might change over time. In addition, a study that compared the effects on fetal testis and adrenal gland after in utero exposure to 500 mg/kg/day DBP demonstrated significantly reduced levels of *StAR* and *CYP11B1* mRNA in the testis with less significant effects on the expression of the same genes in the adrenal gland [29]. Moreover, it is possible that the metabolism and/or distribution in vivo reduces the effect of DBP exposure on adrenal steroids, and higher doses are needed to induce significant changes. It should also be noted that the main stress hormone in humans is cortisol, whereas corticosterone has the corresponding role in rodents [30]. This highlights the importance of complementing animal studies with human models, in particular when studying compounds that affect biological pathways with known species differences, such as steroidogenesis.

Overall, the reduced testosterone levels observed in H295R cells after DBP or MBP exposure are consistent with our recent mouse study [8]. The in vivo results showed that only testosterone was significantly affected, with little or no effect on corticosterone, androstenedione, and progesterone. However, we observed significantly increased levels of testicular CYP11A1, CYP17A1, and HSD3β2, as well as a non-significant increasing trend for HSD17β3 in adult mice one week after five weeks of oral exposure to DBP, as opposed to the decreasing trends observed for the same enzymes in the exposed H295R cells. These differences could, as mentioned above, be due to differences between species or in the dose and duration of the exposure. Furthermore, the biological processes in any organism involve numerous interactions between tissues, cells, and molecules, and it is possible that the observed differences may be due to feedback mechanisms trying to mitigate the reduced testosterone production in mice [8].

To our knowledge, no previous studies have investigated oxidative stress in adrenal cells after DBP or MBP exposure. DBP exposure induced oxidative stress in the H295R cells, illustrated by a significant increase in superoxide generation, together with a small and non-significant increase in protein nitrotyrosine levels. This is in line with our mouse model, where a significant increase of the protein oxidation marker nitrotyrosine was observed one week after five weeks of oral exposure to DBP, even at the lower dose of 10 mg/kg/day [8]. The difference in effect size for protein nitrotyrosine levels is likely due to the difference in exposure times, where the in vivo study was conducted over a much longer period. This indicates that the mechanism behind DBP’s anti-androgenic effects might change depending on the duration of the exposure, where oxidative stress likely plays a more important role in the observed effects in the in vivo study. Since protein oxidation can affect protein activity [31,32,33], the higher nitrotyrosine levels observed in mice could explain how testosterone still decreased despite increased levels of several steroidogenic enzymes. Notably, MBP exposure of H295R cells was found to decrease the protein nitrotyrosine levels. This is in contrast with previous in vivo studies. One study with pubertal rats exposed for three days to 2% MBP in their food observed increased levels of oxidative DNA damage [34]. Another study with adult mice exposed to 200 mg/kg/day MBP for two weeks observed increased levels of testicular ROS and malondialdehyde, a marker for lipid peroxidation [24]. This discrepancy might be due to differences in response between in vitro and in vivo exposure and different sensitivities/mechanisms between cell types. In addition, the decreased nitrotyrosine levels observed in H295R cells exposed to 500 µM MBP is in contrast to the lack of observed effect on superoxide generation. This effect could be due to altered levels of other free radicals that were not measured in this study. It should also be noted that the amount of DHE stained cells in the control was quite low (average 12% of the total number of cells), which might make it difficult to detect decreased superoxide levels, if the effects are subtle. Altogether, these differences observed between DBP and MBP warrants further research to investigate how adrenal and similar steroid producing cells are affected by exposure to phthalates in relation to oxidative stress, in particular in human model systems.

This study confirms the anti-androgenic effects of DBP and MBP in human steroidogenically active cells, as the levels of two androgens, testosterone and androstenedione were reduced after exposure to the phthalates. While this study was done with adrenal cells, the compounds may likely induce similar effects in human Leydig cells, the main producer of testosterone in men [35], as the enzymes catalyzing the different steps to produce testosterone are the same in both tissues, although expressed at different levels [36]. This supports previous epidemiological studies that have linked exposure to DBP with decreased serum testosterone levels [37,38,39,40] and relates to studies observing decreased semen parameters, e.g., reduced sperm motility, in men with higher urinary levels of DBP metabolites [5], as testosterone is vital for initiation and maintenance of spermatogenesis [35].

Although further studies are needed to fully elucidate the mechanism behind the observed effects on steroidogenesis, one possibility is that DBP/MBP interacts with different transcription factors to regulate the expression of steroidogenic enzymes. Activation of the cAMP/PKA pathway, for example, through ACTH stimulation, is the major signaling cascade regulating steroidogenesis [41], and it is possible that the phthalates affect early steps in this cascade, e.g., the binding of the promoter steroidogenic factor-1, to reduce the production of several steroidogenic proteins [42]. Post-translational modifications such as phosphorylation are also vital for the activity of several enzymes in steroidogenesis, and phthalates could potentially affect this as well. The DBP-induced increase in cortisol may be related to post-translational modifications that increase the activity of CYP11B1, in combination with the slight increase in enzyme levels that was observed in the cells.

**Figure 9:**
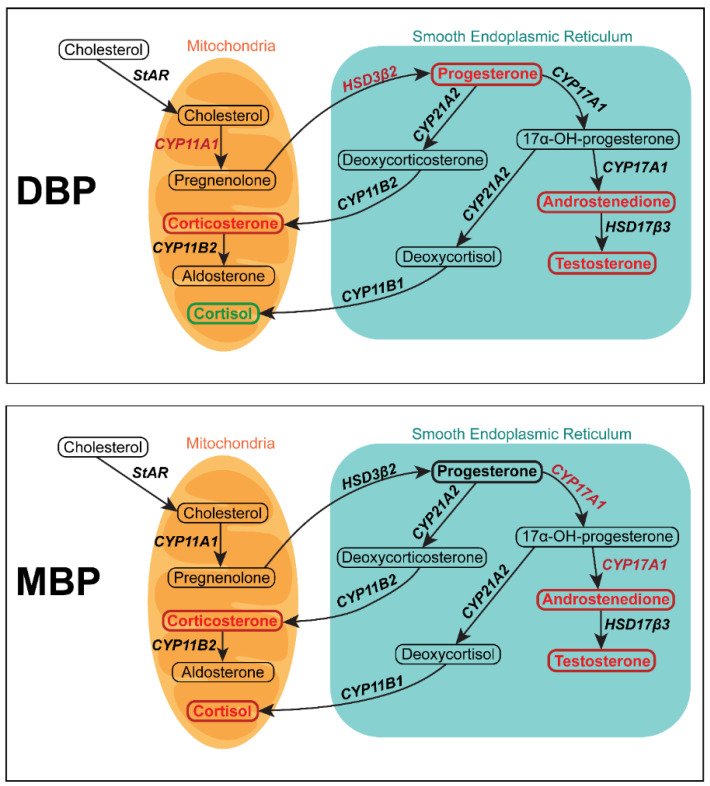
Summary of the effects of DBP (top) and MBP (bottom) exposure on steroidogenesis in H295R cells. Steroid hormones are written inside boxes, and enzymes are written in italic. Hormones and enzymes quantified are marked in **bold**, where red signifies a significant decrease, green a significant increase, and black no significant changes.

To conclude, this study provides a better mechanistic understanding of how DBP and its biologically active metabolite MBP affect steroid hormone biosynthesis in human cells. The findings revealed several differences in mechanism between these phthalates (Figure 9). The DBP-induced effects are generally more consistent with previous in vivo and epidemiological data. DBP was demonstrated to induce more prominent effects on steroidogenesis in human H295R cells compared to MBP. This indicates that, despite the known metabolism of DBP into MBP, the DBP that potentially reaches target tissues can have important effects. Furthermore, the study shows for the first time that DBP can increase cortisol levels in vitro. Meanwhile, MBP induced a cortisol decrease, highlighting differences in their mechanisms of action, which is also reflected in how they alter the levels of key steroidogenic proteins. These findings could have implications for future phthalate research, where more studies that compare the di- and monoester forms are needed to better understand how these differences might be involved in how humans respond to phthalate exposure.

## Figures and Tables

**Table 1:** List of primary antibodies used for western blot analysis.

Antibody	Dilution	Cat. No./Manufacturer
StAR	1:5000	ab133657 Abcam (Cambridge, UK)
CYP11A1	1:1000	ab175408 Abcam
CYP11B1	1:1000	ab171888 Abcam
CYP11B2	1:2000	ab167413 Abcam
CYP17A1	1:2000	ab125022 Abcam
CYP21A2	1:1000	PA5-84152 Invitrogen (Waltham, MA, USA)
HSD3β2	1:1000	ab75710 Abcam
HSD11β2	1:1000	ab203132 Abcam
HSD17β3	1:1000	CF811500 OriGene Technologies, Inc. (Rockville, MD, USA)
Nitrotyrosine	1:2000	05-233 Sigma-Aldrich (St Louis, MO, USA)

## Data Availability

The data presented in this study are available on request.

## References

[1] Giuliani A., Zuccarini M., Cichelli A., Khan H., Reale M. (2020). Critical Review on the Presence of Phthalates in Food and Evidence of Their Biological Impact. Int. J. Environ. Res. Public Health.

[2] Heudorf U., Mersch-Sundermann V., Angerer J. (2007). Phthalates: Toxicology and Exposure. Int. J. Hyg. Environ. Health.

[3] Guo Y., Kannan K. (2012). Challenges Encountered in the Analysis of Phthalate Esters in Foodstuffs and Other Biological Matrices. Anal. Bioanal. Chem..

[4] Kavlock R., Boekelheide K., Chapin R., Cunningham M., Faustman E., Foster P., Golub M., Henderson R., Hinberg I., Little R. (2002). NTP Center for the Evaluation of Risks to Human Reproduction: Phthalates Expert Panel Report on the Reproductive and Developmental Toxicity of Di-n-Butyl Phthalate. Reprod. Toxicol..

[5] Radke E.G., Braun J.M., Meeker J.D., Cooper G.S. (2018). Phthalate Exposure and Male Reproductive Outcomes: A Systematic Review of the Human Epidemiological Evidence. Environ. Int..

[6] Czubacka E., Czerczak S., Kupczewska-Dobecka M.M. (2021). The Overview of Current Evidence on the Reproductive Toxicity of Dibutyl Phthalate. Int. J. Occup. Med. Environ. Health.

[7] Wang J., Zhang X., Li Y., Liu Y., Tao L. (2021). Exposure to Dibutyl Phthalate and Reproductive-Related Outcomes in Animal Models: Evidence from Rodents Study. Front. Physiol..

[8] Källsten L., Almamoun R., Pierozan P., Nylander E., Sdougkou K., Martin J.W., Karlsson O. (2022). Adult Exposure to Di-N-Butyl Phthalate (DBP) Induces Persistent Effects on Testicular Cell Markers and Testosterone Biosynthesis in Mice. Int. J. Mol. Sci..

[9] Chen X., Zhou Q., Leng L., Chen X., Sun Z., Tang N. (2013). Effects of Di(n-Butyl) and Monobutyl Phthalate on Steroidogenesis Pathways in the Murine Leydig Tumor Cell Line MLTC-1. Environ. Toxicol. Pharmacol..

[10] Li L., Chen X., Hu G., Wang S., Xu R., Zhu Q., Li X., Wang M., Lian Q.-Q., Ge R.-S. (2016). Comparison of the Effects of Dibutyl and Monobutyl Phthalates on the Steroidogenesis of Rat Immature Leydig Cells. BioMed Res. Int..

[11] Wang Y.-B., Song L., Cui L.-B., Hong X., Zhang Z.-D., Wang X.-R. (2007). Monobutyl Phthalate Inhibits Steroidogenesis by Downregulating Steroidogenic Acute Regulatory Protein Expression in Mouse Leydig Tumor Cells (MLTC-1). J. Toxicol. Environ. Health Part A.

[12] Frederiksen H., Skakkebaek N.E., Andersson A.-M. (2007). Metabolism of Phthalates in Humans. Mol. Nutr. Food Res..

[13] White R.D., Carter D.E., Earnest D., Mueller J. (1980). Absorption and Metabolism of Three Phthalate Diesters by the Rat Small Intestine. Food Cosmet. Toxicol..

[14] Hallmark N., Walker M., McKinnell C., Mahood I.K., Scott H., Bayne R., Coutts S., Anderson R.A., Greig I., Morris K. (2007). Effects of Monobutyl and Di(n-Butyl) Phthalate in Vitro on Steroidogenesis and Leydig Cell Aggregation in Fetal Testis Explants from the Rat: Comparison with Effects in Vivo in the Fetal Rat and Neonatal Marmoset and in Vitro in the Human. Environ. Health Perspect..

[15] Gazdar A.F., Oie H.K., Shackleton C.H., Chen T.R., Triche T.J., Myers C.E., Chrousos G.P., Brennan M.F., Stein C.A., Rocca R.V.L. (1990). Establishment and Characterization of a Human Adrenocortical Carcinoma Cell Line That Expresses Multiple Pathways of Steroid Biosynthesis. Cancer Res..

[16] Bird I.M., Pasquarette M.M., Rainey W.E., Mason J.I. (1996). Differential Control of 17 Alpha-Hydroxylase and 3 Beta-Hydroxysteroid Dehydrogenase Expression in Human Adrenocortical H295R Cells. J. Clin. Endocrinol. Metab..

[17] Rainey W.E., Bird I.M., Sawetawan C., Hanley N.A., McCarthy J.L., McGee E.A., Wester R., Mason J.I. (1993). Regulation of Human Adrenal Carcinoma Cell (NCI-H295) Production of C19 Steroids. J. Clin. Endocrinol. Metab..

[18] Odermatt A., Strajhar P., Engeli R.T. (2016). Disruption of steroidogenesis: Cell models for mechanistic investigations and as screening tools. J. Steroid Biochem. Mol. Biol..

[19] Duan C., Fang Y., Sun J., Li Z., Wang Q., Bai J., Peng H., Liang J., Gao Z. (2020). Effects of Fast Food Packaging Plasticizers and Their Metabolites on Steroid Hormone Synthesis in H295R Cells. Sci. Total Environ..

[20] Nakajin S., Shinoda S., Ohno S., Nakazawa H., Makino T. (2001). Effect of Phthalate Esters and Alkylphenols on Steroidogenesis in Human Adrenocortical H295R Cells. Environ. Toxicol. Pharmacol..

[21] Jeanneret F., Tonoli D., Rossier M.F., Saugy M., Boccard J., Rudaz S. (2016). Evaluation of Steroidomics by Liquid Chromatography Hyphenated to Mass Spectrometry as a Powerful Analytical Strategy for Measuring Human Steroid Perturbations. J. Chromatogr. A.

[22] Lowry O.H., Rosebrough N.J., Farr A.L., Randall R.J. (1951). Protein Measurement with the Folin Phenol Reagent. J. Biol. Chem..

[23] Aly H.A., Hassan M.H., El-Beshbishy H.A., Alahdal A.M., Osman A.-M.M. (2016). Dibutyl Phthalate Induces Oxidative Stress and Impairs Spermatogenesis in Adult Rats. Toxicol. Ind. Health.

[24] Du J., Xiong D., Zhang Q., Li X., Liu X., You H., Ding S., Yang X., Yuan J. (2017). Mono-Butyl Phthalate-Induced Mouse Testis Injury Is Associated with Oxidative Stress and down-Regulated Expression of *Sox9* and *Dazl*. J. Toxicol. Sci..

[25] Xing Y., Edwards M.A., Ahlem C., Kennedy M., Cohen A., Gomez-Sanchez C.E., Rainey W.E. (2011). The Effects of ACTH on Steroid Metabolomic Profiles in Human Adrenal Cells. J. Endocrinol..

[26] Karlsson O., Rocklöv J., Lehoux A.P., Bergquist J., Rutgersson A., Blunt M.J., Birnbaum L.S. (2021). The Human Exposome and Health in the Anthropocene. Int. J. Epidemiol..

[27] Kim J.H., Lee J., Moon H.-B., Park J., Choi K., Kim S.K., Kim S. (2018). Association of Phthalate Exposures with Urinary Free Cortisol and 8-Hydroxy-2′-Deoxyguanosine in Early Childhood. Sci. Total Environ..

[28] Ahmad S., Sharma S., Afjal M.A., Habib H., Akhter J., Goswami P., Parvez S., Akhtar M., Raisuddin S. (2022). MRNA Expression and Protein-Protein Interaction (PPI) Network Analysis of Adrenal Steroidogenesis in Response to Exposure to Phthalates in Rats. Environ. Toxicol. Pharmacol..

[29] Thompson C.J., Ross S.M., Hensley J., Liu K., Heinze S.C., Young S.S., Gaido K.W. (2005). Differential Steroidogenic Gene Expression in the Fetal Adrenal Gland Versus the Testis and Rapid and Dynamic Response of the Fetal Testis to Di(n-Butyl) Phthalate. Biol. Reprod..

[30] Joëls M., Karst H., Sarabdjitsingh R.A. (2018). The Stressed Brain of Humans and Rodents. Acta Physiol..

[31] Cecarini V., Gee J., Fioretti E., Amici M., Angeletti M., Eleuteri A.M., Keller J.N. (2007). Protein Oxidation and Cellular Homeostasis: Emphasis on Metabolism. Biochim. Biophys. Acta (BBA)-Mol. Cell Res..

[32] Kaur P., Bansal M.P. (2004). Effect of Experimental Oxidative Stress on Steroidogenesis and DNA Damage in Mouse Testis. J. Biomed. Sci..

[33] Zaidi S.K., Shen W.-J., Cortez Y., Bittner S., Bittner A., Arshad S., Huang T.-T., Kraemer F.B., Azhar S. (2021). SOD2 Deficiency-Induced Oxidative Stress Attenuates Steroidogenesis in Mouse Ovarian Granulosa Cells. Mol. Cell. Endocrinol..

[34] Shono T., Taguchi T. (2014). Short-Time Exposure to Mono-n-Butyl Phthalate (MBP)-Induced Oxidative Stress Associated with DNA Damage and the Atrophy of the Testis in Pubertal Rats. Environ. Sci. Pollut. Res..

[35] Sharma R., Agarwal A., Zini A., Agarwal A. (2011). Spermatogenesis: An Overview. Sperm Chromatin: Biological and Clinical Applications in Male Infertility and Assisted Reproduction.

[36] Kempná P., Marti N., Udhane S., Flück C.E. (2015). Regulation of Androgen Biosynthesis—A Short Review and Preliminary Results from the Hyperandrogenic Starvation NCI-H295R Cell Model. Mol. Cell. Endocrinol..

[37] Meeker J.D., Ferguson K.K. (2014). Urinary Phthalate Metabolites Are Associated with Decreased Serum Testosterone in Men, Women, and Children from NHANES 2011–2012. J. Clin. Endocrinol. Metab..

[38] Pan G., Hanaoka T., Yoshimura M., Zhang S., Wang P., Tsukino H., Inoue K., Nakazawa H., Tsugane S., Takahashi K. (2006). Decreased Serum Free Testosterone in Workers Exposed to High Levels of Di-n-Butyl Phthalate (DBP) and Di-2-Ethylhexyl Phthalate (DEHP): A Cross-Sectional Study in China. Environ. Health Perspect..

[39] Pan Y., Jing J., Dong F., Yao Q., Zhang W., Zhang H., Yao B., Dai J. (2015). Association between Phthalate Metabolites and Biomarkers of Reproductive Function in 1066 Chinese Men of Reproductive Age. J. Hazard. Mater..

[40] Woodward M.J., Obsekov V., Jacobson M.H., Kahn L.G., Trasande L. (2020). Phthalates and Sex Steroid Hormones among Men from NHANES, 2013–2016. J. Clin. Endocrinol. Metab..

[41] Stocco D.M., Wang X., Jo Y., Manna P.R. (2005). Multiple Signaling Pathways Regulating Steroidogenesis and Steroidogenic Acute Regulatory Protein Expression: More Complicated than We Thought. Mol. Endocrinol..

[42] Parker K.L., Schimmer B.P. (1997). Steroidogenic Factor 1: A Key Determinant of Endocrine Development and Function. Endocr. Rev..

